# Sarcomas in Adolescents and Young Adults

**DOI:** 10.1007/s11912-026-01781-8

**Published:** 2026-05-20

**Authors:** Jacob R. Greenmyer, Wendy Allen-Rhoades

**Affiliations:** 1 Pediatric Hematology and Oncology, Mayo Clinic, 200 First St. SW, Rochester, MN 55905 USA; 2 Pediatric Hematology and Oncology, Mayo Clinic, 200 First St. SW, Rochester, MN 55905 USA

**Keywords:** Adolescent and young adult (AYA), Sarcoma, Osteosarcoma, Ewing sarcoma, Rhabdomyosarcoma, Non-rhabdomyosarcoma soft tissue sarcoma (NRSTS)

## Abstract

**Purpose of Review:**

This review aims to assess the current state of bone and soft tissue sarcomas (STS) management in the adolescent and young adult population (AYA) with a focus on Ewing sarcoma (EWS), osteosarcoma (OS), rhabdomyosarcoma (RMS), and non-rhabdomyosarcoma soft tissue sarcoma (NRSTS).

**Recent Findings:**

There has been a significant growth in novel agents available for bone sarcomas in the AYA population; trials continue to study the efficacy of these agents. The future of STS will likely include more single histology and targeted biologic studies. Joint pediatric and adult sarcoma clinical trials are feasible.

**Summary:**

AYAs continue to represent an underserved patient population in medical oncology. Collaboration between pediatric and adult practices may increase patient enrollment and enable single histology and biology specific studies. Trials for EWS, OS, RMS, and NRSTS should include both pediatric and adult patients whenever possible.

## Introduction to Adolescent and Young Adult Sarcomas

Adolescents and young adults (AYA), defined by the NCI as patients diagnosed between the ages of 15-39years, are historically underserved in medical oncology. Over the last several decades, AYAs with cancer have experienced modest improvements in survival compared to pediatric and older adult populations [1]. Leveraging the expertise of pediatric and adult oncology is crucial to optimizing sarcoma collaborative research and outcomes for the AYA population.

## Introduction to AYA Bone Sarcomas

Ewing sarcoma (EWS) and osteosarcoma (OS) are the most common primary bone sarcomas in AYAs and account for 3.2% of all cancers in 15- to 24-year-olds [2]. Other AYA bone sarcomas include chondrosarcomas (conventional type or mesenchymal) and exceptionally rare malignancies such as chordoma [2].

## Ewing sarcoma

### Epidemiology

EWS is frequently diagnosed between ages 10–19 years and is the third most common bone sarcoma in all ages [3]). EWS often originates from the extremities, pelvis, ribs, or vertebra, but can arise from soft tissue and viscera [4]. One-quarter of EWS is metastatic at diagnosis [3]. Pathogenic or likely pathogenic germline mutations are present in approximately 13% of patients with EWS and are usually associated with a cancer predisposition syndrome (e.g., Fanconi anemia) or variant in a DNA damage repair gene (e.g., *BRCA1*, *ERCC*, *FANCC*,* POLE*,* RET*) [4].

### Biology

EWS is a small round blue-cell tumor and is characterized by a recurrent balanced chromosomal translocation resulting in the fusion of *EWSR1 *[5]. *EWSR1* is fused with *FLI1* (an ETS transcription factor) in 80% of cases; EWS is less commonly driven by a *EWSR1* fusion with *FEV*, *ETV1*, *ETV4*, or *ERG* [5].

### Prognostic Features

Variables that consistently predict survival in EWS are presence of metastatic disease, histological response to chemotherapy, location of primary tumor (axial EWS have worse overall survival than extremity tumors), and tumor size/volume (tumor size of 8 cm or more is associated with lower overall survival) [6]. Older age tends to be associated with poorer prognosis; however, the age at which patients begin to experience worse prognosis (e.g., 14 years old vs. 18 years old) has not been definitively established [6]. Only 11% of patients on the Children’s Oncology Group (COG) trial, AEWS0031, were aged 18 years or older and the 5-year EFS in this age range was worse than younger patients across arms (47% vs. 72%; *p* < 0.001) [7]. On AEWS1031, age *≥* 18 years was associated with worse overall survival but did report the best event free survival (EFS) compared to historical outcomes; this discrepancy remains unexplained [8].

### Pediatric and AYA Approach to First-line Ewing Sarcoma Therapy

The standard of care chemotherapy for EWS has evolved over the last 40 years through clinical trials that have evaluated permutations of dactinomycin, ifosfamide-etoposide (IE), vincristine-doxorubicin-cyclophosphamide (VDC) [8–17]. The standard of care was established by AEWS0031: a prospective COG trial (2001–2005) that randomized patients < 50 years old with localized EWS to VDC/IE every three weeks (standard) vs. interval compressed VDC/IE every 2 weeks [13]. Compressed VDC/IE had a superior (EFS) compared to the standard arm (73% vs. 65%) with no difference in toxicity [13]. In EuroEwing 2012, VDC/IE led to improved outcomes over the previous European standard VIDE/VAI (induction with vincristine, ifosfamide, doxorubicin, and etoposide; consolidation using vincristine, dactinomycin, with ifosfamide or cyclophosphamide, or busulfan and melphalan) (EFS 67% vs. 61%) with less toxicity [14]. Subsequent studies that added chemotherapeutic agents to VDC/IE have not demonstrated survival benefit; for example, the addition of vincristine-topotecan-cyclophosphamide (VTC) in AEWS1031 did not show survival benefit in patients with nonmetastatic disease [8]. The addition of maintenance chemotherapy to standard of care is not currently recommended outside of a clinical trial [17, 18]. Of note, there is no clear survival benefits for adding IE to therapy for patients with metastatic disease, despite its common use in practice [17].

The duration of therapy (14 vs. 17 cycles) of interval compressed VDC/IE for the treatment of EWS remains controversial. AEWS0031 found a 5-year EFS of 73% with 14 cycles of interval compressed VDC/IE [7]. AEWS1031, a randomized phase 3 trial that evaluated the addition of VTC to interval-compressed VDC/IE for patients with newly diagnosed nonmetastatic Ewing sarcoma, utilized an interval compressed 17-cycle VDC/IE backbone with six additional doses of vincristine. This study did not find a difference in OS or EFS in standard vs. experimental arm and reported a 5-year EFS of 78% for both arms combined [8]. Notably, there was an increased rate of secondary malignancy with 17 cycles (4.3% vs. 2.5%) [8]. To date, no trial has been designed to compare the efficacy of 14 versus 17 cycles of VDC/IE for the treatment of EWS. Gupta and colleagues (2023) published consensus recommendations that recommended 14 cycles over 17 cycles due to the absence of demonstrable benefit and higher likelihood of toxicity with 17 cycles [17]. In practice, many oncologists continue to view the duration of EWS therapy (14 vs. 17 cycles of VDC/IE) as a patient-specific decision that requires nuanced consideration. The National Comprehensive Cancer Network (NCCN) Bone Cancer Version 1.2026 guidelines summarize patient specific considerations for EWS therapy [19].

Long-term follow-up of patients 18 years or older on the interval compressed arm of AEWS0031 demonstrated continued superior EFS (53% vs. 37%) [7]. AEWS1031 included 17% (*n* = 109/642) of patients who were *≥* 18 years old, which was more than prior COG studies (10–14%), and reported a 75% 5-year EFS for patients *≥* 18 years old, which is the highest EFS reported for this patient population [8]. However, there was no difference in survival outcomes between randomized groups in AEWS1031.

Surgical resection with negative margins (R0) is the primary goal for local control of EWS, when possible [19]. The combination of surgery and radiation may be used for large tumors, poor response to neoadjuvant chemotherapy, and positive resection margins [20]. Radiotherapy (45–66 Gy) without surgery is used when surgery would cause unacceptable morbidity [21]. Consolidative radiotherapy is independently associated with improved progression free survival and overall survival in pediatric and AYA patients with metastatic bone and soft tissue sarcomas [22]. Whole lung irradiation (WLI) appears to improve EFS in patients with pulmonary and/or pleural metastases [23].

### Pediatric and AYA Approach to Relapsed/Refractory Ewing Sarcoma

Recurrent EWS occurs in 30–40% of individuals with localized disease and 60–80% with metastatic disease and carries a poor prognosis with less than 25% of patients surviving long term [24]. Time to relapse is an important prognostic factor for relapsed EWS [24]. Stage and sites of relapse also predict outcome; local recurrence (5-year-OS: 35–50%) portends a better outcome than distant-only recurrence (10–20%) and multifocal recurrence (0–10%) [8, 24]. Children younger than 14 years old may have better prognosis than older patients with relapsed EWS.

There is no widely accepted standard of care for relapsed or refractory EWS. The rEECur trial was a recent multi-stage phase 2/3 randomized study that compared outcomes between the following four chemotherapy regimens: (1) gemcitabine, docetaxel, and dexamethasone (GD); (2) irinotecan and temozolomide (IT); (3) single-agent ifosfamide (IFOS); and (4) topotecan and cyclophosphamide (TC) [25–27]. GD and IT arms were terminated at the first and second interim assessments, respectively, due to predicted inferior objective response [25, 26]. IFOS and TC were compared in the phase 3 portion of the study, which calculated a 95% posterior probability that IFOS had improved outcomes over TC (OS 16.8 months vs. 10.4 months; EFS 5.7 months vs. 3.7 months) [27]. rEECur data is limited by differences in the following factors: end point between the phase 3 portion of the study and interim analyses; sample size; time to first recurrence; extent of disease at first recurrence; local control measures; and duration of therapy [17].

A randomized phase 2 trial compared intravenous irinotecan 5-day (higher dose) with vincristine versus intravenous irinotecan 10-day (lower dose) with vincristine [28]. The protracted irinotecan schedule had a higher overall response rate (20.8% vs. 54.5%; *p*=0.019) but there was no difference in progression free survival or overall survival [28]. While 10-day IV irinotecan dosing had lower risk of grade 3/4 gastrointestinal side effects, the scheduling feasibility of this regimen needs to be considered [28]. Oral irinotecan has shown objective responses in both 5-day and 10-day schedules with the 10-day schedule resulting in greater toxicity [29, 30].

The Pediatric MATCH trial found that molecular testing for refractory/relapsed EWS (RR-EWS) revealed an actionable alterations (e.g., *EZH2*,* FGFR1*,* PIK3R1*) in 11.5% (*n* = 12/104) of patients [31]. However, the therapeutic benefit of targeted therapy for EWS has not been established, and the most common loss of function mutations in EWS (*STAG2* and *TP53*) are not yet targetable. Novel therapies continue to be developed for RR-EWS and are at differing stages of research and development (Table 1). Tyrosine kinase inhibitors and cyclin-dependent kinase inhibitors are examples of novel agents that have been investigated for the treatment of RR-EWS as single agents and in combination with traditional chemotherapy backbone (Table 1).

**Table 1:** Summary of selected novel agent AYA studies in Ewing sarcoma and Osteosarcoma

Citation	Trial design	Agent and pharmaceutical class	Eligibility criteria	Number of patients enrolled	Key findings
Ewing Sarcoma					
Choy et al., 2014 [32]	Single-arm, phase II	Olaparib; PARP inhibitor	Metastatic/recurrent EWS, 18–70 years	*N* = 12	PFS 5.7 weeks
Chugh et al., 2020 (SARC025) [33]	Multicenter, phase I	Niraparib (PARP inhibitor) + temozolomide or irinotecan	Advanced EWS, *≥* 13 years	*N* = 29	*Arm 1* PFS: 9 weeks ORR: 0/17 *Arm 2* PFS: 16 weeks ORR: 8%
DuBois et al., 2023 [18]	Randomized, multicenter, phase III	Ganitumab; monoclonal antibody directed towards IGF-1R	Newly diagnosed EWS, < 50 years	*N* = 298	Did not reduce risk of EFS events and may have increased toxicity
Duffaud et al., 2023 (REGOBONE) [34]	Double-blind, placebo-controlled, phase II	Regorafenib; multitargeted TKI	Metastatic relapsed pretreated EWS, *≥* 10 years	*N* = 41	ORR: 22% (*n* = 5/23) PFS: 11.4 weeks with regorafenib vs. 3.9 weeks with placebo
Ludwig et al., 2021 (TK216-01) [35]	Phase II, dose (RP2D)	Tk216 +/- vincristine; EWSR1-FLI1 target	Relapsed/refractory metastatic EWS, mean age 31 years	*N* = 32	CR: 7% SD: 39% PD: 54% SD median duration: 113 days
Meyers et al., 2024 [36]	Multicenter, open-label, phase I/II	TK216; EWS::FLI1 fusion protein antagonist	Relapsed/refractory EWS, ages 11–77	*N* = 85	6-month PFS: 11.9%
Shulman et al., 2023 [37]	Open-label, non-randomized, phase II	Palpociclib (CDK4/6 inhibitor) with ganitumab (IGF-1R monoclonal antibody)	Relapsed EWS, *≥* 12 years	*N* = 10	6-month PFS: 30% CR: 0% PR: 0%
Subbiah et al., 2022 [38]	Open-label, single-arm, Basket phase II	Lurbinectedin; inhibits oncogenic transcription	Pretreated progressive/recurrent EWS, *≥* 18 years	*N* = 28	ORR: 14.3% PFS: 2.7 months
Osteosarcoma					
Davis et al., 2019 (SARC024) [39]	Randomized, double-blind, phase II	Regorafenib; multitargeted TKI	Advanced and metastatic pretreated OST, 18–76 years	*N* = 42	PFS: 3.6 months with regorafenib vs. 1.7 months with placebo
Duffaud et al., 2019 (REGOBONE) [40]	Double-blind, placebo-controlled, phase II	Regorafenib; multitargeted TKI	Progressive pretreated OST, *≥* 10 years old	*N* = 43	PFS: 16.4 weeks with regorafenib vs. 4.1 weeks with placebo
Gaspar et al., 2024 (OLIE) [41]	Randomized, open-label, phase II	Lenvatinib (multitargeted TKI) etoposide, and ifosfamide (LEN-IE)	Relased/refractory OST, 2–25 years	*N* = 40 on experimental arm	PFS: 6.5 months vs. 5.5 months in IE arm
Tang et al., 2024 [42]	Single-arm, exploratory phase II	Camrelizumab (humanized anti-PD-1)	Resectable osteosarcoma, *≥* 14 years	*N* = 64	Amrelizumab combined with neoadjuvant backbone was safe and tolerable
Xie et al., 2019 [43]	Single-arm, phase II	Apatinib; multitargeted TKI	Relapsed/unresectable OST, *≥* 16 years	*N* = 37	ORR: 43% 4-month PFS: 57%
Combined solid tumors including bone sarcomas					
Federico et al., 2020 [44]	Single-arm, phase I	Talazoparib plus irinotecan (arm A) or temozolomide (arm B)	Recurrent/refractory solid tumors, median age 14.6 years	*N* = 41	Arm A ORR: 10% Arm B ORR: 25%
Italiano et al., 2020 (CABONE) [45]	Multi-center, single-arm, phase II	Cabozantinib; multitargeted TKI	Advanced EWS and OST, *≥* 12 years old	EWS: *n* = 39 OST: *n* = 42	EWS: ORR 26%; PFS 4.4 months OST: ORR 12%; PFS achieved in 33% at 6 months
Palmerini et al., 2025 (IMMUNOSARC) [46]	International, single arm, phase II	Sunitinib plus nivolumab; immunotherapy	Advanced and progressing bone sarcomas, 21–74	*N* = 40	6-month PFS: 42%
Schafer et al., 2019 (ADVL1411) [47]	Single-arm, phase I/II	Talazoparib (PARP inhibitor) plus temozolomide	Recurrent/refractory solid tumors, 4–25 years	*N* = 40	2/10 prolonged SD

*EWS* Ewing sarcoma, *OST* osteosarcoma, *CR* complete response, *ORR* objective response rate, *PFS* progression free survival, *PR* partial response, *SD* stable disease, *PD* progressive disease

In summary, there is no standard of care for RR-EWS. At time of relapse, consideration should be given to clinical trial enrollment or selection of a regimen that is informed by factors such as goals of care, individual prognosis, cumulative toxicities, plan for local control, and response.

## Osteosarcoma

### Epidemiology

OS, the most common primary bone tumor in the AYA population, has a peak incidence in late childhood, adolescence, and young adulthood [48]. The male to female ratio is 1.2:1 [48]. Pediatric and AYA OS frequently arises in the extremities, whereas older adult OS most commonly arises in craniofacial and axial locations [48]. Risk factors associated with development of OS include radiation exposure, prior malignancy, and (in older patients) underlying bone disease such as fibrous dysplasia or Paget disease [49]. Most cases of OS are sporadic. However, over one-quarter of patients with OS may carry pathogenic/likely pathogenic variants in cancer-susceptibility genes [49]. The following inherited cancer predisposition syndromes are associated with OS: Diamond-Blackfan anemia, Bloom syndrome, hereditary retinoblastoma, Li-Fraumeni syndrome, Rothmund-Thompson, and Werner syndrome [50].

### Biology

Osteosarcomagenesis is hypothesized to arise from partially differentiated osteoblast precursors [51]. Tumors frequently occur in the metaphasis of long bones in pediatric and AYA populations during times of peak linear growth—a time at which rapidly proliferating bone- and cartilage-producing cells may be at risk of transformation [51]. Osteosarcoma genomics is often characterized by structural rearrangements that are associated with copy number alterations (predominantly copy number loss) of *CDKN2A/B* and *PTEN* and recurrent amplifications of *CCNE1*, *MYC*, and *VEGFA *[51]. Oncogenes and tumor suppressor genes involved in the pathogenesis of OS include *MYC*, *TP53*, and *WWOX *[51].

### Prognostic Features

The following risk factors independently predict OS recurrence: age, histologic response, presence of metastases, primary tumor site, and tumor volume [52]. The histological response of the primary tumor to neoadjuvant chemotherapy is a key prognostic predictor of relapse. Good responders (> 90% necrosis ) have a significantly better five-year overall survival than poor responders (*≥* 10% viable tumor) (overall survival 75–80% vs. 45–55%) [53].

### Pediatric and AYA approach to first-line osteosarcoma therapy

The current standard of care for resectable OS in the AYA population is multidrug neoadjuvant and adjuvant chemotherapy with methotrexate, doxorubicin, and cisplatin (MAP) [53]. All patients with operable OS have a five-year event free survival of 54% and overall survival of 70% [54]. If OS is localized, the five-year EFS and overall survival increase to 60% and 76%, respectively [54]. Dexrazoxane with short infusion doxorubicin provides cardioprotection without compromising tumor response [55]. Standard of care for OS in the AYA population includes removal of pulmonary nodules if feasible. Complete resection of visible pulmonary metastases is necessary for long-term survival in AYA osteosarcoma; aggressive surgical approaches may be needed in curative intent management plans for AYAs that can often tolerate more challenging procedures. Thoracotomy and thoracoscopy are both widely used. AOST2031 is a randomized trial comparing open versus thoracoscopic management of pulmonary metastases in patients with oligometastatic OS (4 or fewer CT identified nodules per lung) (NCT0523516).

Trials that used histologic response to investigate risk-adapted therapy have not demonstrated benefit. Clinical trials intensifying therapy for poor responders have failed to improve outcomes. For example, the addition of ifosfamide and etoposide to MAP (i.e., MAP-IE) in poor responders increased toxicity without improving survival [53]. AOST2032 is an ongoing phase 2/3 study that includes all AYA patients with local and metastatic disease investigating the efficacy of cabozantinib (VEGFR2/MET inhibitor) to MAP therapy (NCT05691478).

### Pediatric and AYA Approach to Relapsed/Refractory Osteosarcoma

Relapse occurs in 40–50% of patients, often within 3 years from diagnosis, and commonly involves the lungs (80%), distant bone (15%), or local site (< 10%) [56]. Complete surgical resection is crucial in patients with relapsed disease and will result in long term remission in 30% of patients [57]. Chemotherapy has historically been used for the treatment of recurrent pre-treated OS; regimens include ifosfamide with or without etoposide, cyclophosphamide, carboplatin, and gemcitabine/docetaxel [58]. Tyrosine kinase inhibitors are used in more recent salvage regimens. Trials for novel therapeutics in relapsed/refractory OS are summarized in Table 1. There is no accepted standard of care, partial and complete responses are rare, and survival benefit is not well established. Clinicians’ experience and patients’ goals of care may contribute to the widespread use of chemotherapy for recurrent pre-treated osteosarcoma, but few studies have investigated an improvement in patient reported quality of life. The NCCN Bone Cancer Version 1.2026 guidelines summarize additional therapies that can be considered for relapsed/refractory OS [19].

## Introduction to Soft Tissue Sarcomas

Soft tissue sarcomas (STS) are a heterogenous group of more than 50 different histologies. STS account for 7–8% of all cancers in individuals < 20 years old, 2–4% in AYAs, and 1–2% in adults [59]. STS in these age groups have unique epidemiology, biology, and management approaches that must be understood as key context for furthering an AYA-specific approach. STS are categorized as rhabdomyosarcoma (RMS) or nonrhabdomyosarcoma soft tissue sarcoma (NRSTS).

## Rhabdomyosarcoma

### Epidemiology

RMS is the most common STS in the pediatric population, accounting for greater than 50% of STS in children and adolescents younger than 15 years [59]. RMS becomes relatively rarer later in life and represents 6.5% and 2% of AYA and adult STS, respectively [60]. RMS is usually sporadic but will occasionally occur in the setting of a cancer predisposition syndrome. In the pediatric population, RMS is more common in Caucasians and males (1.4:1 ratio) [59, 60].

### Biology

There are four major histologic subtypes of RMS: alveolar, embryonal, pleomorphic, and spindle cell sclerosing. The frequency of subtype in adult patients with RMS include pleomorphic (43%), embryonal (34%) and alveolar (23%) [61]. The most common subtypes of RMS in pediatric patients are embryonal (70%) and alveolar (20%) [62]. *PAX-FOXO1* genetics drive aggressive behavior, not morphology, and is often associated with, although not exclusive to, the alveolar histologic subtype of RMS [62]. *PAX-FOXO1* translocation is a balanced translocation of *PAX3* (chromosome 2) or *PAX7* (chromosome 1) with the *FOXO1* gene (chromosome 13) that results in transcription of an onco-fusion protein [62]. Other genetic alterations are increasingly identified in RMS. Mutations in the RAS pathway are identified in over 50% of *PAX-FOXO1* fusion-negative RMS [63–65]. Mutations in *MYOD1* and *TP53* are correlated with inferior outcomes in RMS [63–65]. *MYOD1* is particularly prevalent in RMS at older ages and is usually found in spindle/sclerosing morphology [66]. Pleomorphic histology RMS, more common among adults with RMS, is known for complex genomic alterations including extensive copy-number changes; however, the mechanisms by which these alterations fuel oncogenesis remain incompletely understood [66].

### Prognostic Features and Risk Classification

Risk classification (e.g., low, intermediate, and high risk) incorporates stage, clinical group, patient age, and *FOXO1* fusion status and guides intensity of therapy [65, 67, 68]. The most current COG RMS risk-stratification schemas utilize *PAX-FOXO1* fusion status rather than histology as the main histobiologic prognostic feature [65, 67, 68]. RMS staging (e.g., stage 1–4) is based on site of disease, tumor size, regional nodal involvement, and presence/absence of metastatic disease. Clinical group (e.g., I-IV) is based on localized vs. metastatic disease and completeness of pre-chemotherapy resection [65, 67, 68]. The most significant prognostic features of pediatric RMS are disease extent (e.g., metastatic vs. localized disease) and *PAX-FOXO1* translocation fusion status [63, 66, 68]. Factors that predict improved event-free survival (EFS) in patients with metastatic disease include (1) age 1–9 years, favorable primary tumor site, (2) two or less sites of disease metastasis, and (3) lack of bone or bone marrow metastasis [66].

AYAs with RMS tend to experience worse prognostic features and outcomes than children, even when treated on COG clinical trials. AYAs are more likely than children to present with the following poor prognostic features: clinical lymph node involvement, distant metastatic disease, larger tumors, alveolar histology, and *PAX-FOXO1* translocation [69, 70].

### Treatment: Pediatric-Focused Approach

Multimodal therapy for pediatric RMS includes systemic therapy and local control with surgical resection and/or radiation. The COG standard for low-risk patients is four cycles of vincristine, actinomycin, and cyclophosphamide (VAC) followed by vincristine and dactinomycin (VA) for a total of 24 weeks or 42 weeks of VA [71]. Because superior outcomes in low-risk patients have been achieved (5-year EFS of approximately 90%), current clinical trials are aimed at reducing intensity of systemic therapy to minimize long-term toxicity [68, 71]. ARST2032 is studying the efficacy of VA without cyclophosphamide in patients classified as very low risk [68]. Fourteen cycles of VAC is commonly considered COG standard of care for intermediate-risk COG although there are several regimens that are in use [72]. One upcoming trial concept will comparing higher dose VAC to VAC/VI with maintenance [73]. High-risk RMS (HR-RMS) has a 5-year EFS of approximately 30%. Outcomes for FOXO1 fusion positive metastatic disease is dismal with a 5 year EFS of 6% [67]. COG trial ARST2031 is currently comparing vinorelbine, dactinomycin, and cyclophosphamide (VINO-AC) plus maintenance chemotherapy with vinorelbine and oral cyclophosphamide (VINO-CPO) to VAC plus VINO-CPO maintenance in patients with HR-RMS (closed to accrual, data forthcoming; NCT04994132). ARST0121 and ARST0921 are two of the few randomized clinical trials for relapsed/refractory RMS [74]. NCCN guidelines for RMS are in development.

### Treatment: Adult-Focused Approach

Published studies of older adults with RMS demonstrate inferior outcomes and reflect an increased frequency of the following poor prognostic features: higher rates of distant metastatic disease, larger tumors, unfavorable locations, and unfavorable histology (e.g., pleomorphic histology) [69, 70]. Systemic therapy for adult RMS is less standardized than therapy for pediatric RMS. Variation in adult RMS therapy may be due to the following variables: (1) rarity of RMS in this population, (2) difficulty tolerating chemotherapy, and (3) reduced rates of referral to tertiary care centers [61, 75]. However, some reports suggest that adult RMS has rates of chemosensitivity similar to pediatric RMS despite differences in histologic subtypes and some data to suggests that the inclusion of systemic chemotherapy into treatment regimens for adult RMS results in outcomes similar to those seen in the pediatric population [75]. While there is significant variability in chemotherapy regimens used in adult RMS, most utilize vincristine, doxorubicin, and ifosfamide (VAI) or VAC [61, 75].

### Treatment: Opportunities for a Unified Approach

The rare nature of RMS highlights the need for collaboration among pediatric and adult RMS study groups. There are a number of RMS trials that are open to most/all ages (Table 2). Future clinical trials should be designed to enroll all ages, thereby maximizing the power, generalizability, and tissue banking of these studies. The International Soft Tissue Sarcoma Consortium (INSTRuCT) is a collaboration that unifies the efforts of international pediatric and AYA MRS experts to advance clinical and biologic knowledge [76]. Further exploration of biologic and genomic differences among histologic subtypes may identify targets for novel therapeutics.

**Table 2:** Summary of selected actively recruiting novel agent AYA studies in relapsed and refractory RMS and NRSTS

NCT#	Phase	Agent and Pharmaceutical Class	Eligibility criteria
Rhabdomyosarcoma, chemotherapy and targeted therapy			
NCT06620302	1,2	DT2216 (PROTAC; anti-apoptotic protein B-cell lymphoma-extra-large targeted protein degrader) with irinotecan	Phase 1: 1–21 yo with r/r solid tumors Phase 2: 1–39 yo with DNAJB1:PRKACA fusion
NCT06541262	1,2	Silmitasertib (CK2 inhibitor) with VIT	< 30 yo at initial dx Phase 1: r/r neuroblastoma, EWS, OS, RMS, liposarcoma Phase 2: r/r neuroblastoma, EWS
NCT05634369	2	Universal donor TGFβi NK cells with gemcitabine and docetaxel	1–40 yo 1–4 prior tx for relapse
NCT06709495	1,2	PEEL-224 (Topoisomerase 1 inhibitor), VCR, TMZ	12–49 yo
NCT06721689	1,2	PEEL-224, VCR, TMZ	1–30 yo
NCT05210374	1	Disulfiram (ALDH inhibitor) with copper gluconate and liposomal doxorubicin	> 1 yo
NCT05857969	1	Ex Vivo Drug Sensitivity Testing and Multi-Omics Profiling	≤ 21 yo
NCT06625190	1,2	Alpha/Beta T and B Cell Depletion with Zoledronate	6 momonths-25 years
NCT01804634	2	Haplo HCT with fludarabine, melphalan, and TBI prep and post-transplant CPM, MMF, and shortened tacrolimus duration	1–50 yo
NCT04890093	1,2	PEN-866 (SN-38 conjugated to HSP90 inhibitor) with VCR and temozolomide	12–39 yo
NCT04308330	1	Vorinostat (HDAC inhibitor) with VIT	1–30 yo
NCT04796012	1,2	Atezolizumab (PD-L1 inhibitor) with VIT	6 mo – 30 yo
NCT02508038	1	Alpha/​Beta CD19 + Depleted Haplo Transplant and Zometa	7 mo-21 yo
NCT06589596	1	BGB-58,067 (PRMT5 inhibitor)	≥ 18 yo
NCT04774718	1,2	Alectinib (ALK inhibitor)	Up to 17 yo
NCT06566092	1	Autologous tumor-infiltrating lymphocytes	6 mo – 21 yo, ≥ 8 kg
NCT05329103	1	PEEL-224 (Topoisomerase 1 inhibitor)	≥ 18 yo
NCT04094610	1,2	Oral repotrectinib (TKI against ALK, ROS1, and TRK)	Phase 1: < 12 yo Phase 2: 12–25 yo
NCT04337177	1	Flavored, oral irinotecan with temozolomide	1–30 yo
Rhabdomyosarcoma, CAR-T			
NCT04995003	1	HER2 CAR T cells with nivolumab or pembrolizumab (PD-1 Ab)	1–25 yo
NCT05103631	1	IL-15 armored glypican 3-specific CAR T cells	≥ 18 yo
NCT04377932	1	IL-15 armored glypican 3-specific CAR T cells	1–21 yo
NCT04715191	1	IL-15 and IL-21 armored glypican 3-specific CAR T cells	1–21 yo
NCT06198296	1	IL-15 and IL-21 armored GPC3- CAR T cells	≥ 21 yo
NCT06865664	1	FGFR4 CAR T Cells	3–39 yo
NCT03618381	1	EGFR806 CAR T cells	1–30 yo
NCT04897321	1	B7_H3 CAR-T	0–21 yo
Non-rhabdomyosarcoma soft tissue sarcoma, chemotherapy and targeted therapy			
NCT04851119	1,2	Tegavivint, small molecule Β-catenin inhibitor	Part A: 1–21 yo Part B: 1–30 yo
NCT06620302	1	DT2216 with irinotecan	1–21 yo
NCT06541262	1,2	Silmitasertib (CK2 inhibitor) with VIT	Phase 1: < 30 yo at initial dx
NCT05333458	2	Atezolizumab, selinexor	12 > = yo
NCT05634369	2	Universal donor TGFβi NK cells with gemcitabine and docetaxel	1–40 yo 1–4 prior tx for relapse
NCT06709495	1,2	PEEL-224 (Topoisomerase 1 inhibitor), VCR, TMZ	12–49 yo
NCT05407441	1,2	Tazemetostat (EZH2 inhibitor), Nivo, Ipi	6 months-21 yo
NCT05918640	1,2	Lurbinectedin	≥ 10 yo
NCT05210374	1	Disulfiram (ALDH inhibitor) with copper gluconate and liposomal doxorubicin	> 1 yo
NCT05857969	1	Ex Vivo Drug Sensitivity Testing and Multi-Omics Profiling	≤ 21 yo
NCT06625190	1,2	Alpha/Beta T and B Cell Depletion with Zoledronate	6 months-25 years
NCT01804634	2	Haplo HCT with fludarabine, melphalan, and TBI prep and post-transplant CPM, MMF, and shortened tacrolimus duration	1–50 yo
NCT06083883	1	NK-TCR NY-ESO-1	16–80 yo
NCT06693284	1	Mirdametinib (MEK 1/2 inhibitor) and vorinostat	≥ 12 yo
NCT04332874	2	Pembrolizumab + Isolated Limb Infusion	≥ 12 yo
NCT05985161	2	Selinexor (XPO1 inhibitor)	≥ 12 mo
NCT06239272	1,2	Pazopanib, RT, and Selinexor	≤ 30 yo
NCT05642455	1,2	Afamitresgene Autoleucel (T cell rec tx targeting MAGE-A4 antigen)	2–21 yo, ≥ 10 kg HLA-A*02 positive MAGE-A4 positive
NCT06589596	1	BGB-58,067 (PRMT5 inhibitor)	≥ 18 yo
NCT04774718	1,2	Alectinib (ALK inhibitor)	Up to 17 yo
NCT05919264	1,2	FOG-001 (beta catenin inhibitor)	≥ 18 yo
NCT05329103	1	PEEL-224 (Topoisomerase 1 inhibitor)	≥ 18 yo
NCT04094610	1,2	Oral repotrectinib (TKI against ALK, ROS1, and TRK)	Phase 1: < 12 yo Phase 2: 12–25 yo
Non-rhabdomyosarcoma soft tissue sarcomas, CAR-T			
NCT04995003	1	HER2 CAR T cells with nivolumab or pembrolizumab (PD-1 Ab)	1–25 yo
NCT05103631	1	IL-15 armored glypican 3-specific CAR T cells	≥ 18 yo
NCT04377932	1	IL-15 armored glypican 3-specific CAR T cells	1–21 yo
NCT04715191	1	IL-15 and IL-21 armored glypican 3-specific CAR T cells	1–21 yo
NCT06198296		IL-15 and IL-21 armored GPC3- CAR T cells	≥ 21 yo
NCT03618381		EGFR806 CAR T cells	1–30 yo
NCT04897321		B7_H3 CAR-T	0–21 yo

*ASCT* autologous stem cell transplant, *CAR-T* chimeric antigen receptor T cells, *CPM* cyclophosphamide, *EWS* Ewing sarcoma, *HCT* hematopoietic stem cell transplant, *IVA* ifosfamide, vincristine and actinomycin, *MMF* mycophenolate mofetil, *NK* natural killer, *NRSTS* non-rhabdomyosarcoma soft tissue sarcoma, *OS* osteosarcoma, *RMS* rhabdomyosarcoma, *R/R* relapsed/refractory, *RT* radiotherapy, *TBI* total body irradiation, *TKI* tyrosine kinase inhibitor, *TMZ* temozolomide, *VAC* vincristine, actinomycin, and cyclophosphamide, *VCR* vincristine, *VI* vincristine and irinotecan, *VIT* vincristine, irinotecan, temozolomide, *YO* years old

## Non-rhabdomyosarcoma Soft-tissue Sarcoma

### Epidemiology

NRSTS account for a higher proportion of STS as age increases. NRSTS account for 40% of STS in children < 5 years old and more than 75% of STS in adolescents ages 15–19 [77]. Almost all STS in AYAs and older adults are NRSTS. There are over 50 distinct histologies of NRSTS. The type and frequency of histology is age dependent [77, 78]. Common NRSTS histologies in children include dermatofibrosarcoma (DFS), fibrosarcoma, malignant fibrous histiocytoma (MFS)/undifferentiated pleomorphic sarcoma (UPS), malignant peripheral nerve sheath tumor (MPNST), and malignant fibrous histiocytoma [77]. Histologies commonly seen in the AYA population include DFS, extraosseous EWS, fibrosarcoma, leiomyosarcoma (LMS), liposarcoma (LPS), and MPNST [60]. The histologies most frequently encountered in the adult population include LMS, LPS, and MFH/UPS [59].

### Biology

NRSTS tumor behavior varies based on biology, histology, and age [78–81–82]. Some genomic alterations may be targetable with uniform therapy across multiple tumor types. For example, neurotrophic tyrosine receptor kinase (NTRK)-fusion sarcomas have similar biology that can be exploited independent of tumor type and patient age [81, 82]. By contrast, some tumor behavior can differ significantly based on age, which likely impacts genetic complexity. Desmoid tumors share a common pathobiological dysregulation of the beta-catenin pathway, yet commonly present in different locations in children (e.g., extremity, head, neck) and adults (e.g., abdominal wall, trunk) [79]. Among patients with synovial sarcoma, adults more commonly present with metastatic disease than pediatric patients, despite a shared fusion transcript (t(X;18)(p11.2;q11.2)) [80]. These brief examples demonstrate the heterogeneity of behavior across NRSTS biology, histology, and age landscapes.

### Prognostic Features

Prognostic factors of NRSTS include extent of surgical resection, presence or absence of metastases (i.e., stage), tumor grade, and tumor size. Pediatric and adult treatment approaches differ in their use of these factors. There are nomograms for adult patients with NRSTS that incorporate many patient and disease features and predict outcome [83, 84]. However, similar nomograms have not been developed and widely adopted for pediatric patients [85]. The French Federation of Cancer Centers Sarcoma Group (FNCLCC) system of grading is used for both adults and children, but it is unclear if other grading systems may be more accurate for pediatric patients [86]. While tumor/lymph node/metastasis (TMN) staging is not broadly utilized for pediatric patients, it is used consistently for adults [83–85]. Lymph node spread has historically been treated as metastatic disease. However, more recent investigation has revealed that some histologies with affinity for lymph node spread have relatively good prognosis with multimodality therapy (e.g., angiosarcoma, clear cell sarcoma, epithelioid sarcoma) [87, 88]. Thus, the prognostic impact of lymph node involvement may not be as uniformly significant as once thought [87, 88]. Tumor size cutoffs used for prognostication vary between pediatric and adult practices. Whereas tumor size > 5 cm is considered a poor prognostic feature in pediatric patients, size cutoffs of > 5–8 cm are used for adult patients [89, 90]. Upfront NRSTS adult clinical trials frequently include anatomic site as an eligibility criterion, but anatomic site less frequently impacts the upfront management of NRSTS in pediatric patients [89].

Traditional therapeutic surrogates (e.g., radiographic response) have proven inconsistent in the treatment of NRSTS [91]. Current investigations include the pursuit of predictive, reliable, and sensitive biomarkers of response and outcomes across multiple disciplines including biology, imaging, and pathology [92–94]. While many of these efforts have analyzed pediatric and adult patients and samples separately, this investigation represents an important opportunity for joint investigation that can maximize our understanding of the AYA population.

### Treatment: Pediatric-Focused Approach

INSTRuCT published consensus recommendations for the risk adapted management of pediatric NRSTS [85]. The standard pediatric NRSTS chemotherapy regimen is ifosfamide (3 g/m^2^ per day for 3 days; 9 g/m^2^ per cycle) and doxorubicin (37.5 mg/m^2^ per day for 2 days; 75 mg/m^2^ per cycle) [85]. Pediatric NRSTS radiotherapy dosing ranges from 45 to 59.4 Gy and is based on disease status (e.g., presence of absence of residual disease) and timing (e.g., adjuvant or neoadjuvant) [85]. The survival benefit of adding targeted therapies to the backbone chemoradiotherapy for most pediatric NRSTS has not been demonstrated. However, there are multiple exceptions to these general rules of pediatric NRSTS management. First, certain histologies (such as desmoid-type fibromatosis, desmoplastic small round cell tumor, extraosseous EWS, and rhabdoid tumor) require unique treatment paradigms [79]. Second, particular molecular signatures such as NTRK-fusion sarcomas (e.g., infantile fibrosarcomas) may be treated with targeted monotherapy [82, 95]. Third, cytotoxic chemotherapy should be omitted from the regimen of some histologies (e.g., alveolar soft part sarcoma) due to inherent chemotherapy resistance [85]. Of note, there have been relatively few single-histology pediatric NRSTS studies due to lack of targetable agents and small patient numbers. A comprehensive review of contemporary surgical management of pediatric NRSTS was recently published by Polites and colleagues [96].

### Treatment: Adult-Focused Approach

The management of adult NRSTS is less standardized than pediatric NRSTS [97]. The utilization of neoadjuvant or adjuvant chemotherapy is debated [97]. The management of NRSTS in adults considers location of disease and site-specific nomograms are published for tumors of the extremities and retroperitoneum [83, 84, 98, 99].

Wide surgical resection is the standard of care for localized extremity and truncal NRSTS in adults. Radiation is often included in the therapy of localized disease; however, certain histologies that predominate in adults (e.g., LPS) can be treated with surgery alone [100]. The ideal timing of radiation (e.g., preoperative or postoperative) has not been established, and there are risks and benefits associated with both approaches. Radiation dosing ranges from 44 to 66 Gy [97].

Chemotherapy is considered for high-grade localized disease and large tumors, but there is wide variability in utilization and timing [101]. When chemotherapy is used, the most common regimen is doxorubicin (75 mg/cycle) +/- ifosfamide (7.5–9 g/cycle) [102]). There is some evidence favoring combination therapy, but the efficacy of doxorubicin-ifosfamide over single agent doxorubicin has not been clearly established [103]. Some histology-specific therapies (e.g., trabectedin in myxoid LPS; NY-ESO-1-directed therapy in myxoid/round cell LPS and synovial sarcoma) may be alternatives to standard chemotherapy [104, 105]. However, as a general rule, histology-specific approaches have not been shown to improve outcomes compared to standard chemotherapy. Immunotherapy has demonstrated activity in specific NRSTS histologic subtypes and may be considered in adults with advanced, poor-prognosis disease [105, 106].

### Treatment: Opportunities for a Unified Approach

NRSTS has relatively similar histologies, prognostic features, and therapeutic approaches across the age continuum. These similarities highlight the unique possibility of collaboration between pediatric and adult NRSTS trial groups, which will serve the AYA population. ARST1321, the first NCCN trial to be co-led by a pediatric (COG) and adult consortium (NRG), evaluated the addition of pazopanib to chemoradiation and surgery in children, AYA, and older adults with advanced NRSTS, and demonstrated the feasibility of synchronizing staging, local control, tissue procurement, and systemic therapy for patients of all ages in a clinical trial [107] It seems that the ideal treatment of NRSTS will increasingly move toward single histology and biology-centric approaches [82]. International collaboration between pediatric and adult clinical trial consortiums will be needed to recruit the maximum number of patients. The establishment of consensus definitions of grading, staging, and margin status and harmonization of international clinical trial data will be crucial to the design of meaningful clinical trials and impactful interpretation of data [108] INSTRuCT is facilitating harmonization of international clinical trial data [76, 105, 108]. Further efforts must be made to synchronize data from international pediatric and adult consortiums [109].

## Conclusion

AYAs continue to represent an underserved patient population in medical oncology. While there has been significant growth in novel agents, sarcomas continue to have suboptimal outcomes. Trials including both pediatric and adult patients are feasible. Collaborations between pediatric and adult practices may increase patient enrollment and enable single histology and biology specific studies for STS. Trials for EWS, OST, RMS, and NRSTS should leverage the experience of pediatric and adult oncology whenever possible.

## Key References

- Cash T, Krailo MD, Buxton A, et al. Long-Term Outcomes in Patients with Localized Ewing Sarcoma Treated with Interval-Compressed Chemotherapy on Children’s Oncology Group Study AEWS0031. J Clin Oncol. 2023;41(30): 4724-4728.
  - ⚬ This 10-year follow-up study from AEWS0031 demonstrated that interval-compressed chemotherapy (ICC) resulted in sustained improvements in overall survival and event free survival in patients with localized Ewing sarcoma, without increasing risk for secondary malignancy.
- Wyatt KD, Birz S, Dawkins DS, et al. Creating a data commons: The International Soft Tissue SaRcoma ConsorTium (INSTRuCT). Pediatr Blood Cancer. 2022. 69:e29924.
  - ⚬ This article describes an effort to harmonize international data from patients spanning the pediatric, adolescent, and young adult populations, in an effort to promote key research priorities of rare cancers. The lessons learned from INSTRuCT are translatable to future consortium efforts to study cancers affecting the AYA population.
- Gaspar N, Hung GY, Strauss SJ, et al. Levatinib Plus Ifosfamide and Etoposide in Chidlren and Young Adults with Relapsed Osteosarcoma: A Phase 2 Randomized Clinical Trial. JAMA Oncol. 2024;10(12):1645-1653.
  - ⚬ This phase II international randomized control trial demonstrated the feasibility of early phase collaborative trails for patients with relapsed or refractory osteosarcoma.

## Data Availability

No datasets were generated or analysed during the current study.
